# Identification of diagnostic gene biomarkers related to immune infiltration in patients with idiopathic pulmonary fibrosis based on bioinformatics strategies

**DOI:** 10.3389/fmed.2022.959010

**Published:** 2022-11-24

**Authors:** Xiangdong Dai, Zhihua Yang, Wenjing Zhang, Shuai Liu, Qianru Zhao, Tao Liu, Lu Chen, Lin Li, Yi Wang, Rui Shao

**Affiliations:** ^1^State Key Laboratory of Component-Based Chinese Medicine, Tianjin University of Traditional Chinese Medicine, Tianjin, China; ^2^Institute of Traditional Chinese Medicine, Tianjin University of Traditional Chinese Medicine, Tianjin, China

**Keywords:** idiopathic pulmonary fibrosis, immune infiltration, diagnostic, biomarker, CIBERSORT

## Abstract

**Objective:**

The study aims to identify potential diagnostic markers of idiopathic pulmonary fibrosis (IPF) and analyze the significance of immune cell infiltration in this pathology.

**Materials and methods:**

Download two publicly available gene expression profiles (GSE10667 and GSE24206 datasets) from the GEO database including 48 Idiopathic pulmonary fibrosis (IPF) samples and 21 human control samples and select for distinctly expressed genes (DEG) from them. Lasso regression model and support vector machine recursive feature elimination S,V,R,F analysis were used to check candidate biomarkers. The area under the subject’s work characteristic curve (AUC) value is used to evaluate its recognition ability. The GSE53845 dataset (40 IPF patients and 8 controls) continue to validate the expression level and diagnostic value of biomarkers in IPF. Comprehensive analysis of immune infiltrated cells of IPF was performed using R software and immune cell infiltration estimation analysis tool- deconvolution algorithm (CIBERSORT).

**Results:**

43 DEGs were identified in total. The identified DEGs mostly involve pneumonia, lung disease, collagen disease, obstructive pulmonary disease and other diseases. The activation of IL-17 signaling pathways, amoebic disease, interaction of viral proteins with cytokines and cytokine receptors, protein digestion and absorption, and flaccid hormone signaling pathways in IPF were different from the control group. The expression degree of CRTAC1, COL10A1, COMP, RPS4Y1, IGFL2, NECAB1, SCG5, SLC6A4, and SPP1 in IPF tissue were prominently higher than the normal group. Immune cell infiltration analysis showed that CRTAC1, COL10A1, COMP, IGFL2, NECAB1, SCG5, SLC6A4, and SPP1 were associated with monocytes, plasma cells, neutrophils, and regulatory (treg) T cells.

**Conclusion:**

CRTAC1, COL10A1, COMP, IGFL2, NECAB1, SCG5, SLC6A4, and SPP1 can be used as diagnostic markers for IPF, providing new ideas for the future study of IPF occurrence and molecular mechanisms.

## Introduction

Idiopathic pulmonary fibrosis (IPF) is a progressive mesenchymal lung disease of unknown etiology with common histological and imaging manifestations of interstitial pneumonia (UIP) (1–4). IPF can cause scarring of the lungs, clinically increasing the risk of lung cancer and developing respiratory failure (5). IPF has the characteristics of rapid progression, high fatality rate and poor prognosis, and the median survival after diagnosis in most patients is 2.5 to 3.5 years, with a five-year survival rate of only 20–40% (6). The pathogenesis of IPF has not yet been elucidated, and anti-inflammatory therapy is generally used clinically with glucocorticoids, immunosuppressants, cytotoxic agents or inhibitor (Pirfenidone and Nintedanib) (7–9). With the deepening of clinical research, researchers found that only 20% of IPF patients are sensitive to glucocorticoid therapy, and often transgender reactions(allergies: it is a type of immune reaction, a reaction that occurs after non-peptide drugs are combined with the body’s protein as a hapten to an antigen), there is no specific treatment plan for IPF in the clinic, coupled with the lack of specific clinical manifestations in the early stage of IPF, so the difficulty of diagnosis and treatment is high (10, 11). In recent years, it has been found that IPF often presents familial aggregation, suggesting that it may be a polygenic co-acting diseases. However, there has not been much research on the causative genes of IPF so far. Thus, it is significant to find genes closely related to the pathogenesis of IPF to clarify the pathogenesis of IPF and explore potential drugs for the prevention and treatment of pulmonary fibrosis. And the human Genome project was being completed, omics technology and bioinformatics analysis technology based on high-throughput sequencing/chip analysis have gradually emerged, providing important technical support for the study of the pathological mechanism of complex diseases (12). In order to analyze the differentially expressed genes between normal people and patients, this study used bioinformatics methods to elucidate the signaling pathways and key gene targets closely related to IPF development in the National Biotechnology Center Gene Expression Comprehensive Database GEO,^1^ making the results more accurate and credible.

## Materials and methods

### Data processing

This study used gene chip data from GEO (see text footnote 1) data from the National Biotechnology Information Centre in the United States, serial numbers GSE10667, GSE24206, and GSE53845. Among them, the GSE10667 dataset collected 31 pulmonary fibrosis tissue samples and 15 normal tissue samples, the GSE24206 dataset collected 17 pulmonary fibrotic tissue samples and 6 normal lung tissue samples, and the GSE53845 dataset collected 40 pulmonary fibrosis tissue specimens and 8 healthy lung tissue samples. The Original Data is operated using the Robost Multiarray Averaging function of the oligoR package^2^ and next employ “limma” and “SVA” Two software packages combine two microarray datasets, GSE10667 and GSE24206, into a single dataset as a training dataset. Since the two datasets of GSE10667 and GSE24206 contained samples of IPF tissue and normal lung tissue, they were used for subsequent difference analysis, and the GSE53845 microarray dataset was selected as the model validation dataset for follow-up research.

### Screening for differentially expressed genes

Differentially expressed genes (DEGs) were selected for the combined GSE10667 dataset and GSE24206 dataset using the R language “Limma” and “pheatmap” software packages, and the differences in gene expression were represented by the log2FC of *P* value and fold change (FC). *P* < 0.05 and | log2FC| > 2 were used as thresholds for screening DEGs, and IPF-related differential genes were screened out by volcano maps and heat maps.

### Functional enrichment analysis

Differentially expressed genes were used in the online Database for Annotation (DAVID v6.7)^3^ conducted gene ontology (GO) function and Kyoto encyclopedia of genes and genomes (KEGG) pathway enrichment analysis to comprehensively annotate the biological function information of genes (13). GO functional analysis annotates DEGs from three aspects: biological processes (BP), molecular functions (MF), and cellular components (CC). KEGG pathway enrichment analysis provides high-level pathway function and bioinformatics through large-scale molecular datasets (14, 15). The above analysis can use DAVID to analyze the biological functions of DEGs online, of which *P* < 0.05 is a statistically significant difference. Besides, Disease Ontology (do) enrichment analysis of DEGs using “clusterprofiler” and the DOSE software package in R (16, 17). If *P* < 0.05, the gene set was considered to be significantly enriched.

### Screening of candidate diagnostic biomarkers

To identify major prognostic variables, we use two machine learning algorithms to calculate disease status. Minimum Absolute contraction selection operator (LASSO) is a regression statistics method, which uses regularization way to ameliorate degree of accuracy. Make use of the LASSO in the “glmnet” software package in R to distinguish genes that were prominently relevanted with UC and normal sample discrimination (18). Support vector machines (SVMs) are monitoring machine learning methods that are extensively applied in taxonomy and regression. To avoid overfitting, an RFE algorithm is used to select the optimal gene among the metadata queue. Therefore, in order to discriminate the most recognizable group of genes, we use the support vector machine recursive feature elimination (SVM-RFE) to screen the suitable feature. The nested gene of the two algorithms were fit into and further verify the expression level of candidate genes on the GSE53845 dataset.

### Diagnostic value of characteristic biomarkers for idiopathic pulmonary fibrosis

To examine the predictive value of the identified biomarkers, We used mRNA expression data from 48 IPF and 21 check samples to create ROC curves. The area under ROC curve (AUC) value was used to determine the diagnostic validity of IPF relative to the control sample, and was further validated in the GSE53845 dataset.

### Explore of immunocyte subtypes

The relative scale of infiltrating immunocyte in IPF gene expression profile was quantified, One is called CIBERSORT^4^ was made use of calculating immunocyte infiltration. A reference set containing 22 immunocyte subtypes (LM22) and 1000 permutations was used to estimate the hypothetical immunocyte abundance (19). Relevance analysis and visualization of 22 infiltrative immunocyte with R-pack “corrplot”. Plot violin plots using “vioplot” packets with R format to visualize discrepancy in immunocyte infiltration between IPF samples and check samples.

### Correlation identify between recognized genes and soak immunocyte

The correlation between the recognized gene biomarkers and the level of infiltrative immunocyte was detected using spearman rank relation explored with R. Using charting techniques with “ggplot2” packages, it is possible to visualize the generated associations.

## Results

### Identification of differentially expressed genes in idiopathic pulmonary fibrosis

**FIGURE 1:**
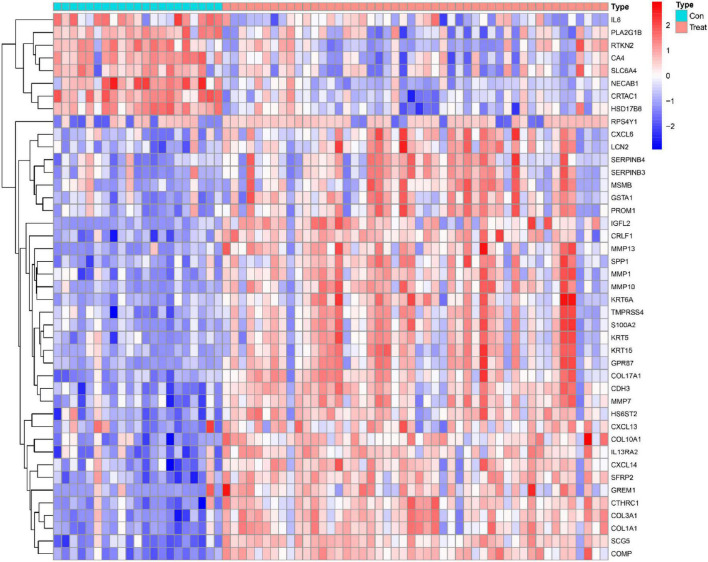
IPF-related genetic heat maps.

**FIGURE 2:**
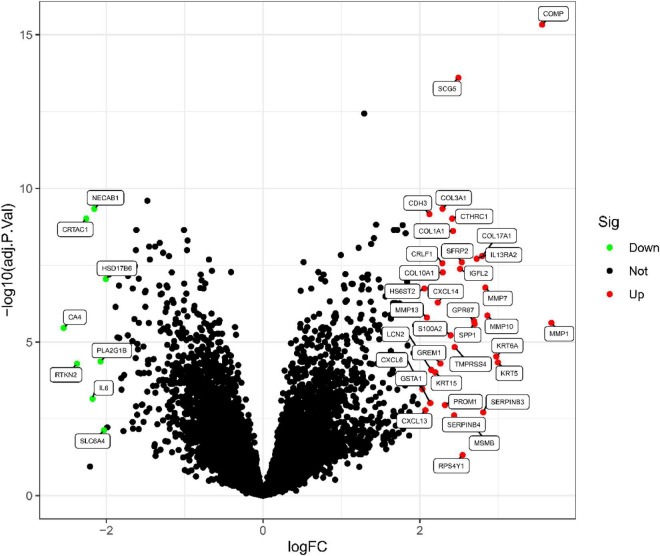
IPF-related differential gene volcanic map.

This study retrospectively analyzed data from 48 IPF and 21 control samples from two GEO datasets (GSE10667 and GSE24206). The LIMMA package is used to perform DEG analysis on the metadata after the batch effect is removed. A total of 43 DEGs are obtained, as shown in Figure 1. 35 genes were remarkably raised and 8 genes were obviously downregulated, as shown in Figure 2.

### Functional correlation analysis

**FIGURE 3:**
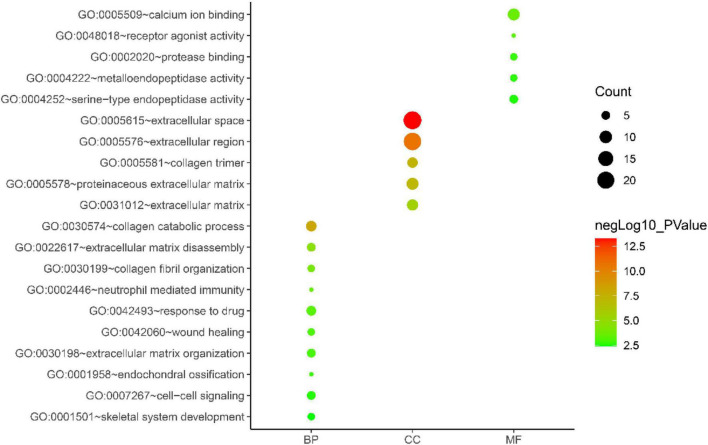
GO enrichment analysis graph.

**FIGURE 4:**
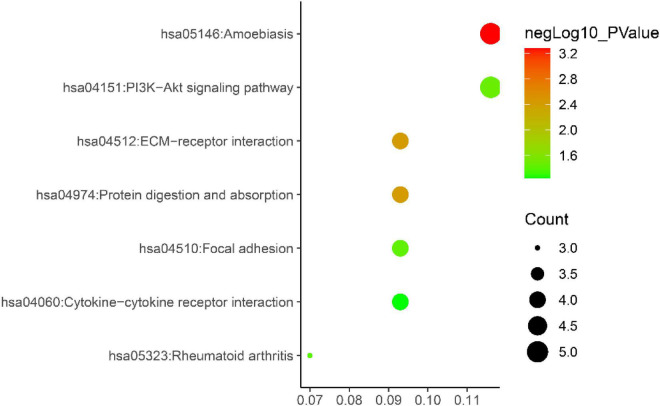
KEGG pathway enrichment diagram.

**FIGURE 5:**
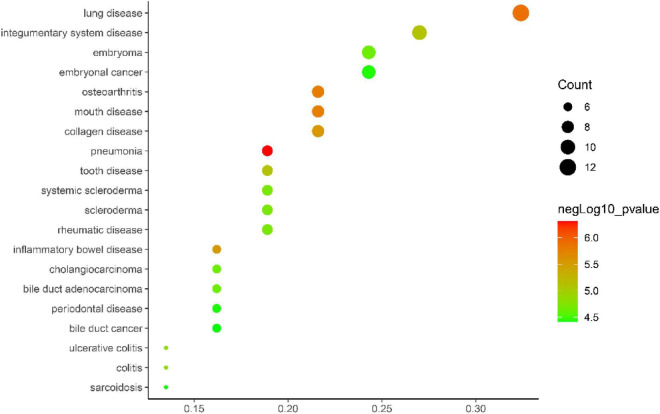
DO enrichment analysis plot.

Gene ontology biological process analysis of DEGs, with *P* < 0.05 as the filter, involving 192 biological processes. The first 20 biological processes include 10 BP, 5 CC, 5 MF, as shown in Figure 3. The KEGG results demonstrated that the enriched pathways mainly involved:ECM-receptor interaction, Amoebiasis, Protein digestion and absorption, PI3K-Akt signaling pathway, Focal adhesion, Rheumatoid arthritis, Cytokine-cytokine receptor interaction, as shown in Figure 4. Conduct pathway enrichment analysis to explore the role of DEGs. The results of functional enrichment demonstrated that diseases enriched by DEGs were mainly related to pneumonia, lung disease, collagen disease, sarcoidosis, osteoarthritis, integumentary system disease, systemic scleroderma, as shown in Figure 5. These results strongly prove that inflammation and immunoreaction plays an vital role in IPF.

### Recognition and confirmation of diagnostic traits biomarkers

**FIGURE 6:**
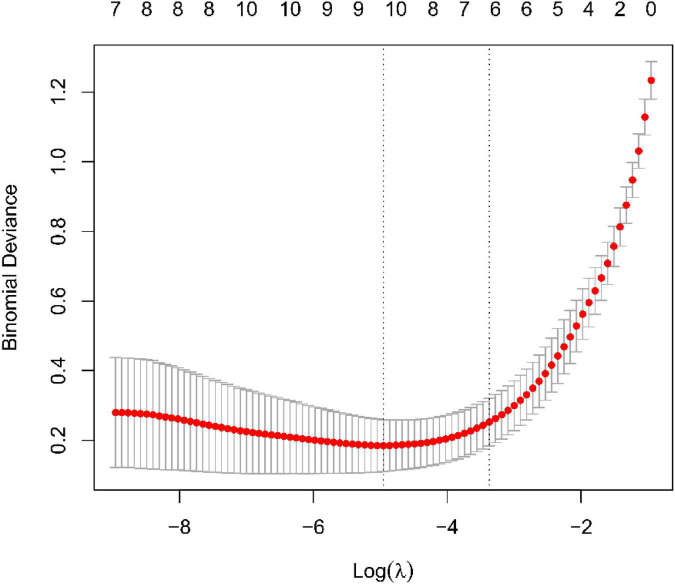
Tuning traits selection in the least absolutem reduction and selection operator model.

**FIGURE 7:**
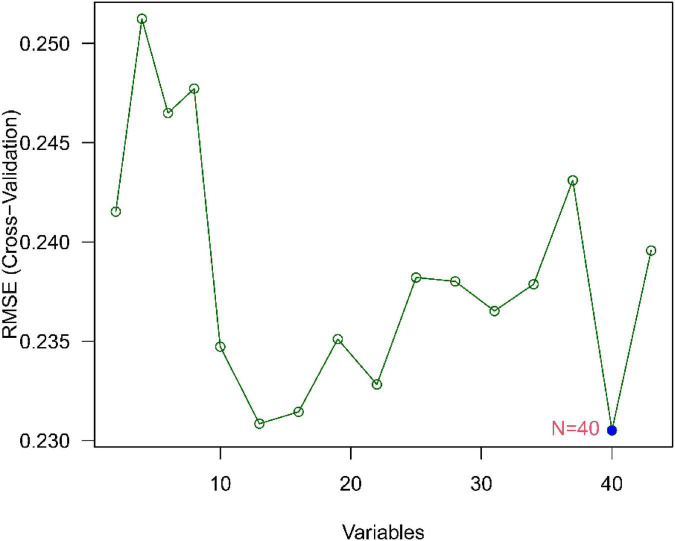
Biomarker selection graph based on support vector machine recursive feature elimination (SVM-RFE) algorithm.

**FIGURE 8:**
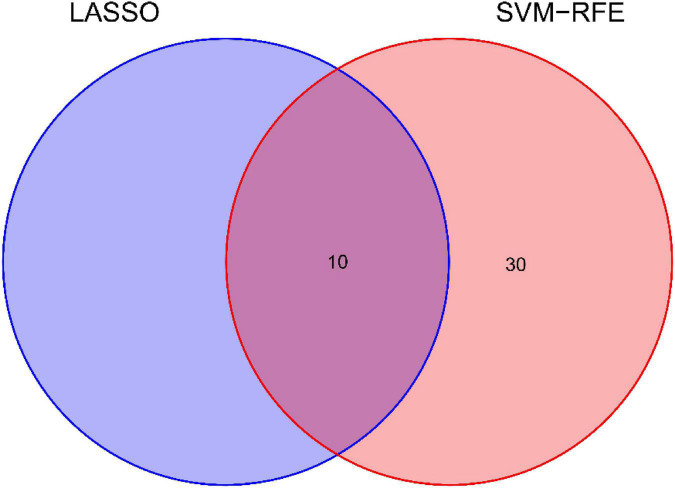
Venn diagram shows the minimum absolute shrinkage and ten diagnostic markers that the selection operator shares with the SVM-RFE algorithm.

**FIGURE 9:**
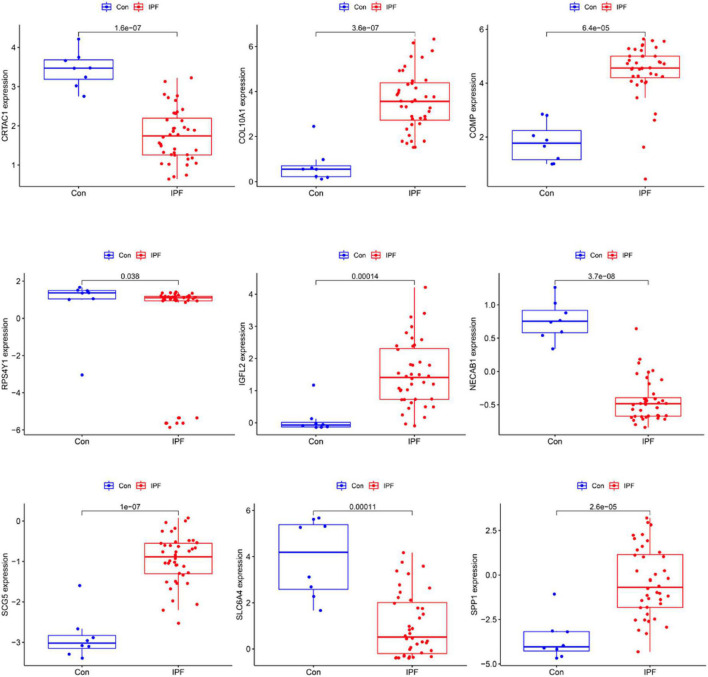
Verification of the level of diagnostic biomarkers in the GSE53845 infomationset.

We use two distinctive algorithms to sifting potential biomarkers. Narrow the DEGs *via* the LASSO returning method to obtain 10 variables as diagnostic biomarkers for IPF, as shown in Figure 6. A subgroup of 40 traits among the DEGs was confirmed using the SVM-RFE, as shown in Figure 7. The 10 superimposed traits (CRTAC1, COL10A1, COMP, RPS4Y1, IGFL2, NECAB1, SCG5, SLC6A4, SPP1, IL-6) between these two algorithms were finally picked as shown in Figure 8. Moreover, to produce more precise and trustworthy outcome, the GSE53845 informationset was used to validate the expression levels of the 10 features. The expression degree of CRTAC1, COL10A1, COMP, RPS4Y1, IGFL2, NECAB1, SCG5, SLC6A4, SPP1 in IPF tissue were prominently higher than the normal group (Figure 9; all *P* < 0.05). Whereas, there was no obviously discrepancies in IL-6 expression between the two groups. Thus, we applied the logistic regression algorithm to build the diagnostic model of nine identified genes in the metadata queue.

### Diagnostic validity of traits biomarkers in idiopathic pulmonary fibrosis

**FIGURE 10:**
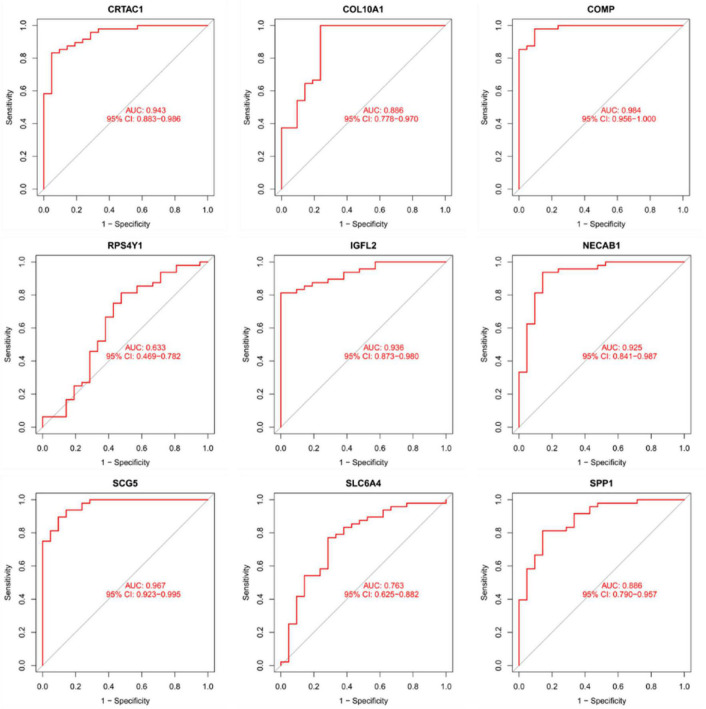
The receiver manipulation characteristic (ROC) curves for diagnostic validity of nine diagnostic markers in the metadata queue.

**FIGURE 11:**
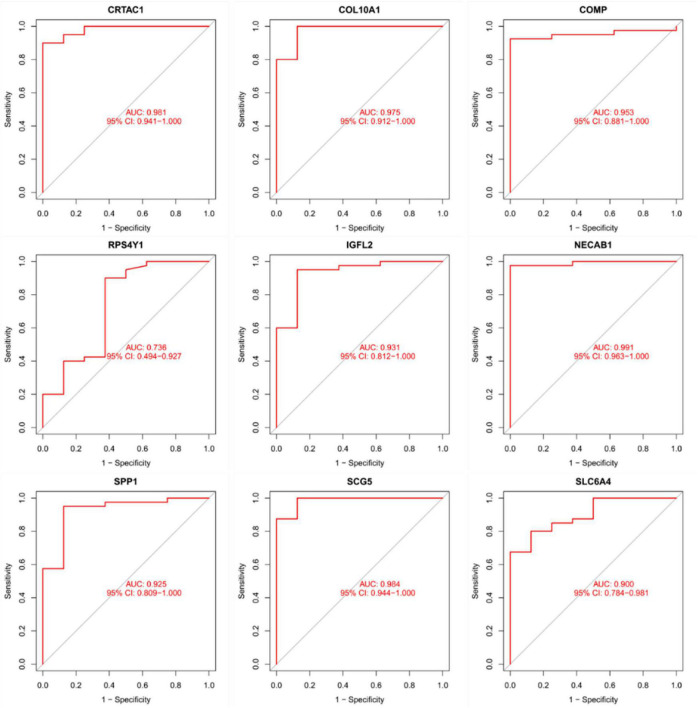
The receiver operating characteristic (ROC) curve of the diagnostic effectiveness of the nine diagnostic markers in the GSE53845 dataset.

As presentation in Figure 10, the diagnostic ability of the three biomarkers in distinguishing IPF from the normal samples showed a advantageous diagnostic value, with an AUC of 0.943 (95% CI 0.883–0.986) in CRTAC1, AUC of 0.886 (95% CI 0.778–0.970) in COL10A1, AUC of 0.984 (95% CI 0.956–1.000) in COMP, AUC of 0.633 (95% CI 0.469–0.782) in RPS4Y1, AUC of 0.936 (95% CI 0.873–0.980) in IGFL2, AUC of 0.925 (95% CI 0.841–0.987) in NECAB1, AUC of 0.967 (95% CI 0.923–0.995) in SCG5, AUC of 0.763 (95% CI 0.625–0.882) in SLC6A4, AUC of 0.886 (95% CI 0.790–0.957) in SPP1. In addition, a strong discrimination ability was confirmed in the GSE53845 dataset with an AUC of 0.981 (95% CI 0.941–1.000) in CRTAC1, AUC of 0.975 (95% CI 0.912–1.000) in COL10A1, AUC of 0.953 (95% CI 0.881–1.000) in COMP, AUC of 0.736 (95% CI 0.494–0.927) in RPS4Y1, AUC of 0.931 (95% CI 0.812–1.000) in IGFL2, AUC of 0.991 (95% CI 0.963–1.000) in NECAB1, AUC of 0.984 (95% CI 0.944–1.000) in SCG5, AUC of 0.900 (95% CI 0.784–0.981) in SLC6A4, AUC of 0.925 (95% CI 0.809–1.000) in SPP1 indicating that the feature biomarkers had a high diagnostic ability, as shown in Figure 11.

### Immunocyte permeability

**FIGURE 12:**
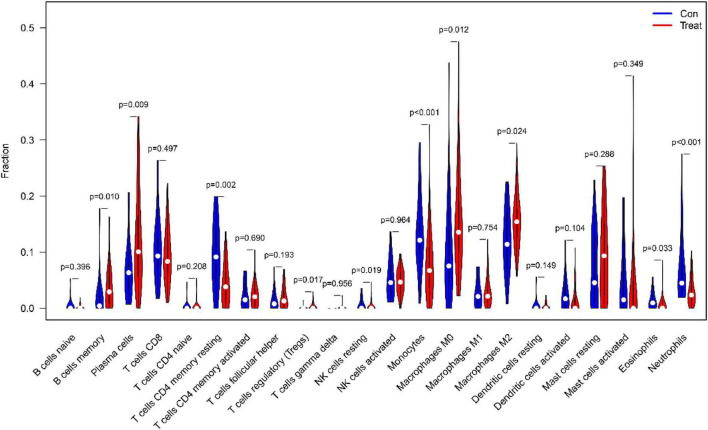
The comparison of 22 immune cell subtypes of IPF tissue with normal tissue green represents normal samples, and red represents IPF samples.

First of all, we investigated the constitution of immunocyte in IPF tissues vs. normal control tissues. The ratio of Plasma cells (*P* = 0.009), regulatory (Tregs) T cells (*P* = 0017), resting NK cells (*P* = 0019), monocytes (*P* < 0.001), M0 macrophages (*P* = 0.012), M2 macrophages (*P* = 0.024), Eosinophils (*P* = 0.033) in IPF tissues were apparently lower than in normal tissues. Nevertheless, the percentage of memory B cells (*P* = 0.010), CD4 memory resting T cells (*P* = 0.001), Neutrophils (*P* < 0.001) in IPF tissues was obviously higher than that in normal tissues, as shown in Figure 12.

**FIGURE 13:**
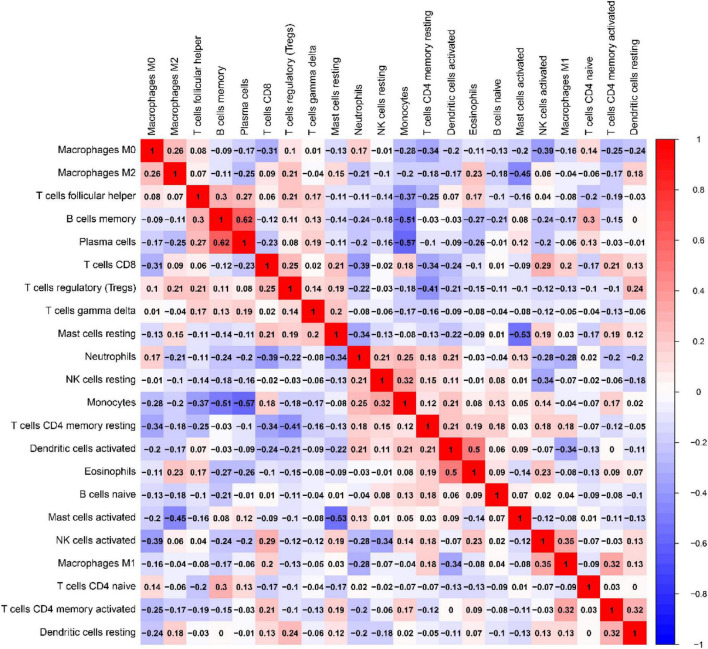
Relevance matrix of components of all 22 immunocyte subtypes. Both the horizontal and Vertical axis show immunocyte subtypes. Immunocyte subtype composition (higher, lower, and the same correlated levels are shown in red, blue, and white, respectively).

Correlation of ratio of each immunocyte in IPF tissue samples as presentation in Figure 13. In UC tissues, immune cells that make up a larger than correlation factor include: negatively correlated visible monocytes and Plasma cells (−0.57), memory B cells (−0.57), follicular helper T cells (−0.37); activated mast cells and static mast cells (−0.53), M2 macrophages (−0.53); activated NK cells and M0 macrophages (−0.39), resting NK cells (−0.34); CD4 memory resting T cells and follicular helper T cells (−0.41), M0 macrophages (−0.34), and CD8 T cells (−0.34); CD8 T cells and M0 macrophages (−0.31); M1 macrophages and activated dendritic cells (−0.34). The above negative correlation suggests a relationship between these immune cells in the process of IPF disease. Positive correlations are visible memory B cells with Plasma cells (0.62), follicular helper T cells(0.30); monocytes and resting NK cells (0.32); Eosinophils and activated dendritic cells(0.50); T cells CD4 native and memory B cells (0.30); M1 macrophages and activated NK cells(0.35); CD4 memory activated T cells and M1 macrophages (0.32), activated dendritic cells (0.32). The above positive correlation suggests that these immune cells have a synergistic relationship between these immune cells in the course of IPF disease.

### Correlation analysis between the eight biomarkers and infiltrating immune cells

**FIGURE 14:**
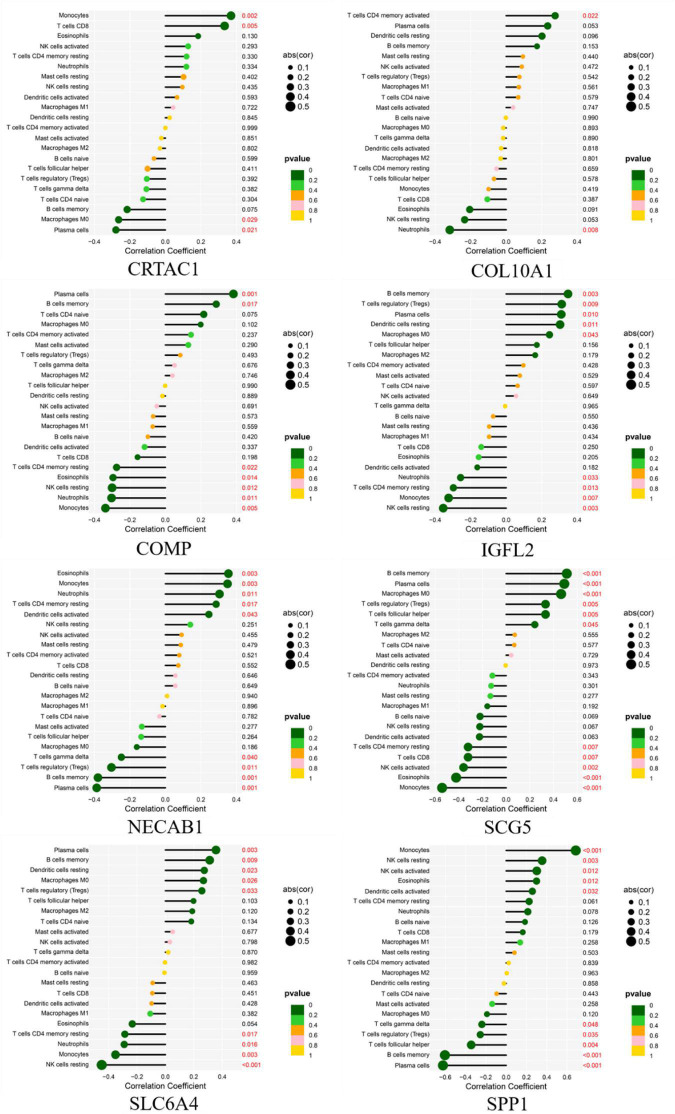
Correlation between diagnostic gene biomarkers and infiltrating immune cells in IPF.

As shown in Figure 14, CRTAC1 was positively correlated with Monocytes (R = 0.37, *p* = 0.0018), T cells CD8 (R = 0.33, *p* = 0.0053) and negatively correlated with Plasma cells (R = −0.28, *p* = 0.021), Macrophages M0 (R = −0.26, *p* = 0.029). COL10A1 was positively correlated with T cells CD4 memory activated (R = 0.28, *p* = 0.022), and negatively correlated with Neutrophils (R = −0.32, *p* = 0.008). COMP was positively correlated with Plasma cells (R = 0.38, *p* = 0.0012), B cells memory (R = 0.29, *p* = 0.017) and negatively correlated with Monocytes (R = −0.34, *p* = 0.0046), Neutrophils (R = −0.3, *p* = 0.011), NK cells resting (R = −0.3, *p* = 0.012), Eosinophils (R = −0.29, *p* = 0.014), T cells CD4 memory resting (R = −0.27, *p* = 0.022).IGFL2 was positively correlated with B cells memory (R = 0.35, *p* = 0.0033), T cells regulatory (Tregs) (R = 0.31, *p* = 0.0089), Plasma cells (R = 0.31, *p* = 0.010), Dendritic cells resting (R = 0.3, *p* = 0.011), Macrophages M0 (R = 0.24, *p* = 0.043) and negatively correlated with NK cells resting (R = −0.35, *p* = 0.003), Monocytes (R = −0.32, *p* = 0.007), T cells CD4 memory resting (R = −0.3, *p* = 0.013), Neutrophils (R = −0.26, *p* = 0.033). NECAB1 was positively correlated with Eosinophils (R = 0.35, *p* = 0.003), Monocytes (R = 0.35, *p* = 0.003), Neutrophils (R = 0.3, *p* = 0.011), T cells CD4 memory resting (R = 0.29, *p* = 0.017), Dendritic cells activated (R = 0.24, *p* = 0.043) and negatively correlated with Plasma cells (R = −0.39, *p* = 0.001), B cells memory (R = −0.38, *p* = 0.001), T cells regulatory (Tregs) (R = −0.3, *p* = 0.011), T cells gamma delta (R = −0.25, *p* = 0.040). SCG5 was positively related to Plasma cells (R = 0.36, *p* = 0.003), B cells memory (R = 0.31, *p* = 0.009), Dendritic cells resting (R = 0.27, *p* = 0.023), Macrophages M0 (R = 0.27, *p* = 0.026), T cells regulatory (Tregs) (R = 0.26, *p* = 0.033) and negatively correlated with NK cells resting (R = −0.45, *p* < 0.001), Monocytes (R = −0.35, *p* = 0.003), Neutrophils (R = −0.29, *p* = 0.016), T cells CD4 memory resting (R = −0.29, *p* = 0.017). SLC6A4 was positively correlated with Monocytes (R = 0.69, *p* < 0.001), NK cells resting (R = 0.35, *p* = 0.003), NK cells activated (R = 0.3, *p* = 0.012), Eosinophils (R = 0.3, *p* = 0.012), Dendritic cells activated (R = 0.26, *p* = 0.032) and negatively correlated with Plasma cells (R = −0.62, *p* < 0.001), B cells memory (R = −0.6, *p* < 0.001), T cells follicular helper (R = −0.34, *p* = 0.004), T cells regulatory (Tregs) (R = −0.25, *p* = 0.035), T cells gamma delta(R = −0.24, *p* = 0.048). SPP1 was positively correlated with B cells memory (R = 0.51, *p* < 0.001), Plasma cells (R = 0.49, *p* < 0.001), Macrophages M0 (R = 0.46, *p* < 0.001), T cells regulatory (Tregs) (R = 0.33, *p* = 0.005), T cells follicular helper (R = 0.33, *p* = 0.005), T cells gamma delta (R = 0.24, *p* = 0.045) and negatively related to Monocytes (R = −0.54, *p* < 0.001), Eosinophils (R = −0.42, *p* < 0.001), NK cells activated (R = −0.36, *p* = 0.002), T cells CD8 (R = −0.32, *p* = 0.007), T cells CD4 memory resting (R = −0.32, *p* = 0.007).

## Discussion

Idiopathic pulmonary fibrosis is a rare, progressive, and often lethal type of chronic fibrotic interstitial lung illness (2). IPF is disabling, the mortality rate is high, the etiology and pathogenesis are not clear, the prognosis is not ideal, and improving the treatment effect is still a serious challenge (2). Therefore, in-depth understanding of the pathogenesis of the disease can further discover new treatments to improve the prognosis of patients. Bioinformatics is an emerging interdisciplinary discipline that uses tools such as mathematics, computer science, and biology to analyze and study biological data in order to understand the biological significance of the data. Recent period, bioinformatics are widely used in the study of nosogenesis, which can find genes that play a major role in the development of diseases, so as to discover new pathogenic targets of diseases, which is of great significance for understanding the development system of diseases and creating novel treatment methods.

In this study, the raw data of the two datasets of GSE10667 and GSE24206 were collected from the GEO database and the datasets were comprehensively analyzed. Altogether 43 DEGs were selected, of which 35 were highly expressed in IPF and 8 DEGs were lowly expressed in IPF. Subsequently, DEGs were analyzed for GO function, KEGG enrichment and disease enrichment, which were consistent with the results of previous mechanism studies, and the biological functions of DEGs were mainly concentrated in cell adhesion, biological adhesion, collagen metabolism, cytoskeletal development, extracellular matrix changes, cytokine interactions, etc. The results of KEGG pathway enrichment analysis show that DEGs are mainly related to ECM receptor interactions, protein digestion and absorption, PI3K-Akt signaling pathway, and cytokine–cytokine receptor mutual influence and other signaling pathways. The results of disease enrichment analysis showed that DEGs were mainly involved in diseases such as pneumonia, lung disease, collagen disease, sarcoidosis, osteoarthritis, integumentary system disease, systemic scleroderma, and so on.

Based on the LASSO regression model and SVM-RFE analysis, COMP, SPP1, SLC6A4, COL10A1, CRTAC1, IGFL2, NECAB1, and SCG5 were identified as diagnostic markers of IPF. TGF-β1 is generally believed to be a key cytokine driving fibrosis (20), and the expression level of TGF-β1 in most fibrotic tissues is significantly increased. Cartilage oligomeric matrix protein (COMP) is a non-collagenous glycoprotein component of extracellular matrix (ECMs) that accentuates TGF-β1 signaling and is associated with extracellular matrix polymerization and stiffness. COMP is a biomarker associated with the severity of pulmonary fibrosis in systemic sclerosis (21). The expression level of COMP in patients with IPF was significantly increased, and the increase in serum COMP expression level was closely related to the decrease of the patient’s lung capacity index (22). Secretory phosphoprotein 1(SPP1) is a protein formerly related to pulmonary fibrosis and COPD in lung process in mice (23). Recent years, it has been found out that SPP1 expression has been upregulated in bleomycin-mediated models of pulmonary interstitial fibrosis (24, 25), mouse models of asbestos lungs (19), and irradiation-induced models of interstitial fibrosis (26), suggesting that SPP1 may have played a certain regulatory role in the progression of pulmonary fibrosis. Single-cell RNA sequencing has been shown to show that the macrophage subset in IPF has high expression of SPP1 (27), and the expression of SPP1 gradually increases as inflammation and fibrosis deepen during fibrosis (24). SPP1 result in human fibrotic lung disease, and rising in SPP1 are related to IPF (28). Lamothe et al. (29) shows Spp1 and Sirpa were identified as key conserved genes in the regulation of smoking and pulmonary fibrosis in humans and mice. SLC6A4 is a serotonin transporter gene whose expression or methylation is strongly associated with the onset, phenotype, and prognosis of depression (30, 31). At the same time, the SLC6A4 variant was associated with low survival in colorectal cancer patients (32). In addition, SLC6A4 variants are a risk factor for coprous obstructive pulmonary disease with lung cancer (33). However, we have not found that SLC6A4 has been involved in pulmonary fibrosis. Studies confirm that the progression of the disease of IPF is accompanied by severe collagen metabolism disorders (34). COL1A1, COL1A2, COL8A1, COL10A1, and COL14A1 are members of the collagen family. During the pathogenesis of pulmonary fibrosis, a large amount of extracellular matrix is deposited in the lungs and is highly expressed in the lung tissues of IPF. Type I collagen is the main lung collagen and contains two alpha1 chains (COL1A1) and one alpha2 chain (COL1A2). IPF pathology is manifested by early thickening of the alveolar wall and alveolitis of death in epithelial cells, alterations that eventually lead to the formation of pulmonary fibrosis, characterized by overexpression of type I collagen (21). Type I and type III collagen play a major role in the development of fibrosis lungs. In the IPF, both type I and type III collagen production increased (35). However, we have not found that the characteristic gene COL10A1 identified in this study has been involved in pulmonary fibrosis. CRTAC1, regarded as an opponent of nogo receptor-1 (36). IL-1β and TNF-α in patients with osteoarthritis can induce and obviously upregulate the expression of CRT in articular chondroblasts or synovial fibroblasts (37), CRTAC1 can be used as a biomarker to distinguish chondrocytes from osteoblasts and mesenchymal stem cells (38). At present, there are no reports on CRTAC1 and pulmonary fibrosis at home and abroad. In addition, no other potential markers IGFL2, NECAB1, SCG5 were found to play a role in pulmonary fibrosis. This study uses bioinformatics to screen out differentially expressed genes, some of which have been confirmed to be involved in IPF and some of which have not yet been studied, suggesting that these genes may be new research targets for the study of the pathogenesis of IPF.

The categories of immunocyte penetration in IPF and normal samples were evaluate using CIBERSOTR. The results showed that various immunocyte subtypes were closely related to the vital biological processes of IPF. An increased permeability of Plasma cells, regulatory (Tregs) T cells, resting NK cells, Monocytes, M0 macrophages, M2 macrophages, and Eosinophils, and a decreased penetration of memory B cells, CD4 memory resting T cells, and neutrophils has been found to be associated with the onset and development of IPF. Moreover, by utilizing correlation analysis between COMP, SPP1, SLC6A4, COL10A1, CRTAC1, IGFL2, NECAB1, SCG5 and immune cells, COMP, SPP1, SLC6A4, COL10A1, CRTAC1, IGFL2, NECAB1, and SCG5 were found to be correlated with Plasma cells, monocytes, Neutrophils, regulatory (Tregs) T cells. Chronic inflammation and the immune response is significantly important in the progression of the IPF (39), Immune response was thought to be related to IPF (40). Clinical studies have confirmed that the level of monocytes was closely related to IPF mortality. A retrospective, multicentre cohort study (41) showed that patients with IPF with higher monocyte counts were at higher risk for poor outcomes. In addition, Kreuter et al. (42) In patients with IPF, an elevated monocyte count has been reported to be related to an increased risk of IPF progression, hospitalization, and mortality. Monocyte counts can be included in the clinical evaluation of patients with IPF but detailed future research was required to assess this (42, 43). Studies confirmed (44) that the neutrophil inhibitor civirolox sodium reduces the degree of pulmonary fibrosis by inhibiting TGF-β expression; In addition, Gregory et al. (45) used bleomycin to induce fibrosis in mice, and found that fibroblasts and myofibroblasts in mice with NE(-/-) were greatly reduced, and the degree of pulmonary fibrosis was less than in the normal group, further confirming that neutrophils are participated in the immune adjustment of IPF. Moreover, clinical studies (46) have also confirmed that neutrophil levels are closely related to IPF mortality, and that neutrophil recruitment into the bronchoalveolar cavity is considered a predictor of early death in patients with IPF (47). Clinical studies have also found that BAL neutrophils and eosinophilia in IPF patients are directly related to CCL18 concentrations, and their mechanism may be related to the production of reactive oxygen species by neutrophils and the participation of eosinophils in the formation of inflammatory injury in pulmonary fibrosis (48). Normally, very few lymphocytes are present in the alveolar interstitium, while lymphocytes in the lungs of patients with IPF are significantly elevated. Among them, T lymphocytes are the main effector cells: Th1 type cells express antifibrosis factors, while Th2 type cells express profibrosis factors, Th1/Th2 imbalance is one of the pathogenesis of pulmonary fibrosis, Treg cells participate in irradiation-induced pulmonary fibrosis by facilitating fibroblast aggregation, weakening the Th17 reaction and governing the Th1/Th2 equilibrium (49). Regulatory T-cells (Tregs) are also involved in the pathogenesis of IPF. Boveda-Ruiz et al. (50) found that Tregs increase the release of TGF-β1 and collagen deposition in the early stages of pulmonary fibrosis, demonstrating that Tregs have a pro-fibrosis effect in the early stages; However, Tregs in the late stage have an anti-fibrotic effect: Xiong et al. (49) studies have shown that the depletion of Tregs can increase Th17 cell expression, thereby prompting the Th1/Th2 balance to shift to Th1, thereby reducing the degree of pulmonary fibrosis. Studies have shown that Tregs can also reduce fibroblast aggregation and reduce the degree of pulmonary fibrosis by inhibiting fibroblast-9 and chemokine ligand 12 (51, 52). M2 macrophages can secrete IL-10, chemokines CCL18 and other profiblination cytokines, such as CCL18 levels have increased significantly in the patient’s serum, BALF and AM culture, indicating that it can promote collagen production by lung fibroblasts and play an important role in inflammatory immune response (53). Therefore, selective activation of macrophages is crucial in IPF development.

Large-scale studies of high-throughput sequencing techniques and molecular mechanisms have provided clues to the origin and development of IPF, but further research is needed to elucidate the pathogenesis of IPF. Considering the potential for false-positive results, limited sample sizes, and potential heterogeneity in the analysis of a single microarray dataset, we have integrated data from two datasets (GSE10667 and GSE24206), and the molecular mechanisms of IPF pathogenesis involved in the obtained DEGs still need to be further explored. The limitation of this study is that although the DEGs associated with IPF were extracted through data mining and detailed bioinformatics analysis of multiple datasets, and the diagnostic characteristic genes of IPF were obtained according to THE LASO regression model and SVM-RFE analysis, it was still necessary to perform related experiments for example western blotting and immunohistochemical analysis to verify the level of these genes in IPF. In addition to identifying IPF-associated DEGs and characteristic genes by analyzing two datasets, more reliable and accurate DGEs need to be explored through new techniques and bioinformatics analysis methods. In addition, further loss of function and function must be carried out *in vivo* and *in vitro* to obtain experimental confirmation. This study illustrates a trustworthy and comprehensive perspective on the pathogenesis and progress of IPF, and a large number of potential molecules relevant to the etiopathogenesis of IPF can be aquired through bioinformatics analysis, and can be verified by later experiments to provide a more detailed basis for the diagnosis and treatment of IPF.

## Conclusion

In a word, CRTAC1, COL10A1, COMP, IGFL2, NECAB1, SCG5, SLC6A4, and SPP1 were identified as diagnostic biomarkers of IPF. Monocytes, Plasma cells, Neutrophils, Regulatory (Tregs) T cells, M2 macrophages, Eosinophils, and CD4 memory resting T cells are tightly relevant to the appearance and progress of IPF. These immune cells may be developed as targets for immunotherapy in patients with IPF. In summary, these research have shown that neutrophils are related to the formation of IPF pulmonary fibrosis, and through in-depth study of the immune regulation mechanism of sextrophils in IPF, IPF target therapeutic drugs can be developed.

## Data availability statement

The datasets presented in this study can be found in online repositories. The names of the repository/repositories and accession number(s) can be found in the article/supplementary material.

## Author contributions

RS and YW conceived the study. XD and ZY wrote the manuscript. WZ, SL, QZ, and TL edited the pictures. LC and LL revised the manuscript. All authors read and approved the final manuscript.

## Footnotes

1
https://www.ncbi.nlm.nih.gov/geo/

2
http://www.bioconductor.org/packages/release/bioc/html/oligo.html

3
https://david.ncifcrf.gov/summary.jsp

4
https://cibersortx.stanford.edu/
